# A multi-phase structured cascade model for mass training of community healthcare workers in performing clinical breast exams in remote regions

**DOI:** 10.7189/jogh.14.04255

**Published:** 2024-12-20

**Authors:** Taleaa Masroor, Russell S Martins, Aiman Arif, Talaiha Chughtai, Falak Madhani, Nida Zahid, Sana Zeeshan, Lubna Vohra, Salima Khan, Mishal Hidayat, Hamna Amir, Sajid Bashir Soofi, Abida K Sattar

**Affiliations:** 1 Department of Surgery, John Hopkins University, Baltimore, Maryland, USA; 2 Division of Thoracic Surgery, Department of Surgery, Hackensack Meridian School of Medicine, Hackensack Meridian Health Network, Edison, New Jersey, USA; 3 Aga Khan University, Karachi, Pakistan; 4 Aga Khan Health Services, Karachi, Pakistan

## Abstract

**Background:**

Clinical breast exam (CBE) by outreach healthcare workers (HCW) may help downstage breast cancer in resource-limited areas where mammography may not be feasible. We evaluated the effectiveness of a pilot cascade-model training programme for HCWs in remote areas of Pakistan.

**Methods:**

The training programme comprised three phases. In phase one, fellowship-trained breast surgeons at a metropolitan academic centre trained six HCWs to perform CBEs. In phase two, these six HCWs (master trainers) trained 15 additional HCWs, implementing cascade training. In phase three, the consultant breast surgeon conducted a re-evaluation and refresher course for all 21 HCWs at least one year after the original training session. We assessed CBE ability and skills through pre- and post-changes through self-reported confidence and direct observation of procedural skills.

**Results:**

Significant improvements in learners’ self-reported confidence and CBE skills were observed in both phases one and two. The median scores in the learners’ post-training self-reported confidence and CBE skills (inspection, palpation, and lymph node examination) improved by 20% and 46.2%, respectively, indicating excellent learning outcomes of the cascade training sessions. Phase three showed sustained high scores in self-reported confidence and CBE skills more than one year later.

**Conclusions:**

Mass training of outreach HCWs in remote regions in performing CBE may be possible with a structured multiphase cascade-training model and may be an important step in downstaging symptomatic breast cancer in low-resource settings.

Early diagnosis is fundamental to the timely and effective management of breast cancer, and nationwide screening programmes have significantly reduced mortality rates in high-income countries [1]. However, implementing mass screening interventions is extremely challenging in low-resource settings, as such programmes require technical expertise (mammographers and radiologists) and adequate infrastructure [2–4]. In their position paper on mammography screening, the World Health Organization (WHO) explicitly states that mammography screening is not cost-effective for low- and middle-income countries (LMICs). The focus of available resources should be on early diagnosis of women with symptomatic lesions to ensure universal access to effective diagnosis and treatment [2]. This early detection of symptomatic lesions is most effectively done using physician-performed clinical breast examinations (CBE), as it has been reported to detect up to 45% of lesions missed by mammography (i.e. false negatives) [5–7]. This allows the opportunity for earlier management initiation and better outcomes. Such is the value of a systematically performed CBE, which the WHO also states should be considered a low-cost screening tool in resource-constrained settings with limited access to specialised testing [2]. Pakistan is an LMIC in Southeast Asia with one of the highest age-standardised incidence rates of breast cancer in the region [8]. The logistical challenges and resource constraints endemic to the healthcare systems of most LMICs also severely limit the practicality and sustainability of conventional mass screening programmes for breast cancer, particularly in remote regions without access to healthcare facilities. In addition, the physician-to-patient ratio in remote regions of the country is significantly lower than the WHO standard, thus also limiting the feasibility of physician-performed CBE for early detection of symptomatic breast lesions [9]. Lastly, the conservative and patriarchal nature of society in Pakistan may also restrict women from participating in breast cancer detection programmes that require them to travel to a healthcare facility. These factors result in almost half of women diagnosed with breast cancer in Pakistan having locally advanced or metastatic disease, leading to a poor prognosis [10].

Community outreach programmes have been very successful in several aspects of healthcare in Pakistan, particularly maternal and child health [11,12]. Our group at the Aga Khan University in Pakistan has implemented similar home outreach programmes for the community-wide early detection of symptomatic breast lesions in rural areas of the Sindh province, with largely encouraging early results [13]. However, the sustainable implementation of such a programme in more geographically remote and inaccessible rural regions is severely limited by the inability of trained healthcare personnel to visit the area routinely. In this study, we present our experience of a cascade model for training outreach healthcare personnel for community-wide early detection of symptomatic breast lesions. We implemented a cascade-training model, whereby we trained healthcare personnel to successfully impart their CBE skills to novice personnel downstream and educate them regarding breast cancer, attempting to leverage the existing local healthcare workforce in remote regions. The use of CBE in our study is based upon the WHO recommendation regarding its appropriateness in low-resource settings such as ours [2]. In this study, we aimed to demonstrate a model which can be replicated to perform mass-training of many healthcare workers (HCWs) via a snowball effect, hence exponentially increasing the reach of the programme.

## METHODS

### Study design and setting

We performed this educational interventional study using a pre-and post-test design to evaluate the impact of breast cancer-related knowledge and CBE skills training intervention from December 2021 to May 2023. The study team was primarily located at the Aga Khan University (AKU) in Karachi, Pakistan. AKU is an academic tertiary care private hospital, and Karachi is the largest metropolitan city in the country. The field site for this study was in the Gilgit-Baltistan-Chitral (GBC) region of Pakistan, which is located over 2000 km north of Karachi. This region is marked by mountainous terrain, making it geographically challenging to access. The target area in this region spanned 12 districts covering a total of approximately 63 500 km^2^ and a population of over 1.2 million.

### Study population

The study population consisted of female HCWs native to the GBC regions. These HCWs included both physician and non-physician HCWs, such as lady health visitors (LHVs) and nurses. The minimum qualification to become an LHV is eight years of primary and secondary school education.

### Study flow

#### Phase one: training of the master trainers

We identified and invited to participate in the study six experienced HCWs (three physicians, two nurses, and one LHV) from various districts of GBC interested in breast health and who previously demonstrated interest in teaching. These six HCWs were to later serve as trainers of other HCWs in phase two, i.e. as master trainers (MTs). The education, training, and evaluation strategies in phase one have been detailed in a subsequent section of the methodology. The education and training in phase one were imparted by fellowship-trained breast surgeons at AKU over five days.

#### Phase two: cascade training

The six HCWs trained in phase one were responsible for the cascade/down-stream training of 15 HCWs across various districts of GBC. The education, training, and assessment employed were identical to phase one and were conducted at various health camps in the Gilgit region over three days.

#### Phase three: one-year re-evaluation and refresher course

One year after the initiation of phase two (17 months after training MTs and 14 months after cascade training), we assessed the CBE skills of conveniently available participants from GBC regions via direct observation of procedural skills (DOPS) of CBE. This training and assessment were conducted on consenting patients who visited the ‘breast camps’ in the Gilgit region, conducted by a fellowship-trained breast surgeon over three days to assess the long-term retention of CBE skills. Following this assessment, a refresher course was conducted for the HCWs using the previously established curriculum.

### Data collection

We collected participants’ demographic data (level of education, years of experience, employment details, and involvement in previous breast cancer-related education/training) and data on the participants’ knowledge regarding breast cancer and self-reported confidence in performing CBE. We prepared a 14-item questionnaire (including questions regarding risk factors for breast cancer, signs and symptoms of breast cancer, common myths regarding breast cancer, and self-reported confidence in performing CBE (Appendix S1 in the **Online Supplementary Document**) using elements from a previous tool and was validated for the appropriateness of its content by three fellowship-trained breast surgeons [14]. Prior to the commencement of the study, the questionnaire was piloted and statistically validated on 20 HCWs, not including any study participants, from our hospital in Karachi, Pakistan. The Cronbach’s alpha coefficient was 0.71 for knowledge assessment, which falls within the acceptable range. This tool was administered before and after the educational/training intervention.

### Direct observation of procedural skills for CBE

This assessment tool was adapted from the previous literature [15] to assess CBE technique, including inspection, palpation, and lymph node (LN) examination and HCWs’ skills in detecting and interpreting findings (Appendix S2 in the **Online Supplementary Document**). The three fellowship-trained breast surgeons served as independent examiners for the DOPS of CBE. The correct performance of each step/component of the CBE was awarded a point. There was no negative marking. The DOPS of CBE was performed before and after the educational/training intervention, and its inter-rater reliability was always above 80%.

### Content and structure of the educational/training intervention

We designed the curriculum for the educational/training intervention based on literature and recommendations made by the American Cancer Society. Knowledge and awareness training included education regarding the risk factors, signs and symptoms, and basic breast cancer management. It also included information about common myths regarding breast cancer and strategies for how to dispel these myths within the general population. In addition, we coached HCWs on how best to educate the general population regarding breast cancer, especially with regard to the life-saving potential of early identification and initiation of treatment. This aspect of the curriculum was delivered using didactic lectures, printed informational material, and educational videos. The learners were provided ongoing access to all educational materials.

CBE training consisted of the demonstration of the correct performance of a CBE by a fellowship-trained breast surgeon (live in phase one and through a recorded video in phase two*)*, followed by hands-on practice on a simulated breast model and subsequently on consenting patients in a real-world clinical setting. There was special emphasis on correctly performing every step of the CBE, detecting lumps greater than two cm and other suspicious breast abnormalities, documenting findings using a standardised template, and interpreting positive findings to determine the need for appropriate referral for further evaluation. All participants continue to have access to the CBE video and didactic materials. HCWs were also coached on how to serve as educators and trainers for breast-related knowledge/awareness and CBE training, enabling them to serve as MTs in the future and perpetuating a sustainable model of cascade training.

The study curriculum was delivered using a combination of basic English and Urdu. English and Urdu are the co-official languages of Pakistan and are generally taught across schools in the country, including the GBC regions. Urdu is also the lingua franca of Pakistan. All the HCWs included in our study had intermediate-advanced proficiency in English and Urdu and comprehended all the educational material and assessment tools.

### Statistical analysis

We performed statistical analysis using the Statistical Package for Social Sciences, version 23 (International Business Machines Corporation, Armonk, New York, USA). Breast cancer-related knowledge was expressed numerically as a percentage of correct responses. The total score of the DOPS of CBE was also calculated as a percentage and averaged across the three independent examiners to provide a final percentage score for CBE performance. Numeric variables were described using median (MD) and interquartile range (IQR), and categorical variables were described using frequencies and percentages. The difference between pre-scores and post-scores was calculated as a change in scores (post-score minus pre-score equals the change in score) and presented as an MD change (MD and IQR). We compared the paired numeric data using the Wilcoxon signed-rank test, and unpaired numeric data using the Mann-Whitney U test. Our analysis was designed to sequentially explore whether: 1) learning was effective across both phases one and two, 2) learning was retained one year later in phase three, and 3) learning was comparable between physicians vs. non-physician HCWs and between employees of government institutions vs. private institutions. We considered a *P*-value <0 · 05 significant for all analyses.

## RESULTS

### Characteristics of the HCWs

Out of the 21 HCWs included in this study, approximately half (47.6%) were LHVs, with the remaining being physicians (38.1%) or nurses (14.3%). Only two (9.6%) had prior formal education or training in breast health. Just over half (52.4%) had previous experience in raising breast cancer awareness within a community (**Table 1**).

**Table 1:** Healthcare worker characteristics

Variable	n (%)
Highest level of education	
*High-school diploma*	11 (52.4)
*Bachelor’s degree or higher*	10 (47.6)
Professional experience in years	
*0–5*	8 (38.1)
*6–10*	6 (28.6)
*>10*	7 (33.3)
Type of healthcare worker	
*Lady health visitor*	10 (47.6)
*Nurse*	3 (14.3)
*Doctor*	8 (38.1)
Previous education/training in breast health	
*Trained to perform CBE*	1 (4.8)
*Attended a course about breast cancer*	1 (4.8)
Employed by	
*Aga Khan Health Service Pakistan*	12 (57.1)
*Government of Pakistan*	8 (38.1)
*Not reported*	1 (4.8)
Prior experience of raising breast cancer awareness	11 (52.4)

CBE – clinical breast exam

### Outcomes of phase one, phase two (cascade training), and phase three (one year post training)

The educational and training intervention delivered by the fellowship-trained breast surgeon resulted in statistically significant improvements in the six HCWs’ breast cancer-related knowledge and confidence in performing CBE (**Table 2**). Statistically significant improvements were also seen on DOPS of CBE overall (pre-test MD = 62.4, IQR = 46.7, 69.4; post-test MD = 89.3, IQR = 83.1, 95.0), and in the LN examination and palpation components of CBE. An increase of 20.8% was also seen in the inspection component of CBE, although this did not achieve statistical significance. The MD time taken to perform CBE decreased significantly from 270 (IQR = 180.0, 300.0) seconds in the pre-test to 150 seconds (IQR = 150.0, 180.0) during the post-test.

**Table 2:** Pre- and post-comparisons of healthcare workers

Variable	Pre-scores, MD (IQR)	Post-scores, MD (IQR)	Difference, MD (IQR)*	*P*-value
Phase 1: training of MTs by breast surgeons†				
*Knowledge/awareness*	65.4 (42.4, 80.8)	92.3 (73.1, 100.0)	19.3 (13.4, 40.4)	0.028
*Confidence*	40.0 (35.0, 65.0)	80.0 (80.0, 80.0)	40.0 (15.0, 45.0)	0.039
*CBE skills*	62.4 (467.0, 69.4)	89.3 (83.1, 95.0)	23.9 (18.6, 45.0)	0.028
*Inspection*	62.5 (37.5, 100.0)	97.2 (80.6, 100.0)	20.8 (0.0, 50.0)	0.068
*LN examination*	66.6 (49.2, 70.8)	88.9 (80.6, 95.8)	20.0 (13.9, 40.3)	0.028
*Palpation*	66.7 (66.7, 66.7)	100.0 (100.0, 100.0)	33.3 (33.3, 33.3)	0.014
*Time taken in seconds*	270.0 (180.0, 300.0)	150.0 (150.0, 180.0)	–105.0 (22.5, 150.0)	0.042
Phase 2: training of other HCWs by MTs (cascade training)†				
*Knowledge/awareness*	61.5 (53.8, 69.2)	69.2 (53.8, 84.6)	7.7 (0.0, 15.4)	0.005
*Confidence*	60.0 (60.0, 80.0)	100.0 (80.0, 100.0)	40.0 (20.0, 20.0)	0.002
*CBE skills*	45.5 (27.2, 63.6)	100.0 (90.0, 100.0)	46.2 (36.3, 63.7)	0.001
*Inspection*	100.0 (50.0, 100.0)	100.0 (100.0, 100.0)	0.0 (0.0, 50.0)	0.160
*LN examination*	16.7 (16.7, 66.7)	100.0 (100.0, 100.0)	66.7 (16.7, 83.3)	0.001
*Palpation*	50.0 (0.0, 100.0)	100.0 (100.0, 100.0)	50.0 (0.0, 100.0)	0.014
*Time taken in seconds*	120.0 (60.0, 156.0)	190.0 (150.0, 210.0)	24.0 (0.0, 120.0)	0.012

CBE – clinical breast exam, HCWs – healthcare workers, IQR – interquartile range, LN – lymph node, MD – median, MT – master trainers

*Difference denotes median change between the pre- and post-scores.

†Presented as % unless specified otherwise.

The educational and training intervention delivered by the six MTs to the subsequent batch of 15 HCWs also resulted in statistically significant improvements in the HCWs’ breast cancer-related knowledge (pre-test MD = 61.5, IQR = 53.8, 69.2; post-test MD = 69.2, IQR = 53.8, 84.6) and confidence in performing CBE (pre-test MD = 60.0, IQR = 60.0, 80.0; post-test MD = 100 (IQR = 80.0, 100.0). Statistically significant improvements were also seen in the DOPS of CBE overall (pre-test MD = 45.5, IQR = 27.2, 63.6; post-test MD = 100.0, IQR = 90.0, 100.0) and in the LN examination and palpation components of CBE. No MD increase was observed in the inspection component of CBE, as both the pre-and post-scores were 100% for that component. Contrary to phase one, the MD time taken to perform CBE increased significantly (pre-test MD = 120.0, 60.0, 156.0; post-test MD = 190.0, IQR = 150.0, 210.0). The learning outcomes of phases one and two are shown in **Table 2**.

Finally, 13 (61.9%) of the HCWs who were conveniently available for reassessment demonstrated sustained or increased competency in CBE skills, with the MD score for DOPS of CBE overall and all its components being 100%. The MD time to perform a CBE (300 seconds) was significantly higher than the immediate post-training times in phases one and two.

### Comparisons across pooled (learners from phases one and two) cohorts of HCWs

Across both phases one and two (n = 21), non-physician HCWs had significantly more significant improvement in DOPS of CBE overall (54.6% vs. 31.8%, *P* = 0.010) and the LN examination component of CBE (83.3% vs. 16.7%, *P* = 0.010), compared to physicians. The time taken to perform CBE increased significantly for HCWs working in government hospitals but remained unchanged for those working in private hospitals. (**Table 3****,** **Table 4**).

**Table 3:** Score change comparisons between physician and non-physician HCWs

Change*	Physician, MD (IQR)	Non-physician, MD (IQR)	*P*-value
Knowledge	15.4 (15.3, 28.9)	7.7 (3.9, 19.2)	0.104
Confidence	20.0 (20.0, 35.0)	20.0 (20.0, 40.0)	0.860
Overall CBE	31.8 (17.2, 37.5)	54.6 (36.4, 69.7)	0.010†
Inspection	0.0 (0.0, 24.9)	19.4 (0.0, 50.0)	0.595
LN examination	16.7 (13.7, 33.3)	83.3 (25.6, 83.3)	0.030†
Palpation	33.3 (0.0, 83.3)	33.3 (0.0, 75.0)	0.916
Time taken in seconds	0.0 (–67.5, 0.0)	100.0 (–30.0, –130.0)	0.140

CBE – clinical breast exam, LN – lymph node, IQR – interquartile range, MD – median

*Change presents post-score minus pre-score. Presented as % unless specified otherwise.

†Statistically significant.

**Table 4:** Score change comparisons of between private vs. government HCWs

Change*	Private, MD (IQR)	Government, MD (IQR)	*P*-value
Knowledge	15.3 (0.0, 23.1)	11.6 (7.7, 21.1)	0.697
Confidence	40.0 (20.0, 40.0)	20.0 (20.0, 20.0)	0.104
Overall CBE	27.3 (21.1, 59.1)	50.4 (38.7, 84.1)	0.064
Inspection	0.0 (0.0, 27.8)	25.0 (0.0, 8.5)	0.500
LN examination	33.3 (15.4, 80.6)	58.3 (16.7, 83.3)	0.456
Palpation	33.3 (0.0, 33.3)	75.0 (0.0, 100.0)	0.238
Change in time taken in seconds	0.0 (–105.0, 60.0)	62.0 (2.8, 135.0)	0.037†

CBE – clinical breast exam, IQR – interquartile range, LN – lymph node, MD – median

*Change presents post-score minus pre-score. Presented as % unless specified otherwise.

†Statistically significant.

## DISCUSSION

In this study, we demonstrated the feasibility of a structured multiphase cascade model for the education and training of HCWs in performing CBE to identify breast abnormalities warranting further evaluation and referral. In phase one, we successfully trained six HCWs to serve as MTs to subsequent batches of trainees. In phase two, these six MTs were able to train 15 HCWs to perform a CBE successfully. Our one-year follow-up demonstrated excellent retention of CBE skills, further lending credibility to the feasibility and effectiveness of the cascade model. In addition, the educational curricula delivered in both phase one and phase two significantly improved the awareness of breast cancer-related knowledge among HCWs, better equipping them to educate the community and dispel myths and misinformation.

Almost two-thirds of Pakistan’s population, totalling over 159 million people, reside in rural areas of the country with limited healthcare access and a low physician-to-population ratio [16]. Many of these rural areas are further constrained by geographical inaccessibility, making outreach interventions from external regions challenging and unsustainable. Thus, the most optimal and sustainable solution to providing high-quality primary healthcare in these rural and remote regions is to identify, empower, and support existing healthcare resources. Several studies from LMICs and rural settings have shown the effectiveness of training HCWs to improve women’s knowledge regarding breast cancer and increase the number of early diagnoses [17–19]. Our group at AKU has also had experience implementing large-scale community-wide home outreach-based symptomatic breast abnormality detection interventions in a rural country region [13]. The current study provides proof-of-concept for implementing a more sustainable solution for the early detection of symptomatic breast abnormalities that are particularly relevant to geographically remote rural regions. All 21 HCWs included in our study were native to the GBC regions and highly motivated towards making a meaningful impact in their communities. The group of HCWs included both physicians and non-physicians from the public and private healthcare sectors. A greater improvement in CBE skills was observed amongst non-physician HCWs compared to physicians. These results demonstrate the generalisability of the educational interventions to healthcare professionals across a wide range of training and backgrounds and the ability to empower non-physician HCWs to perform CBE with sufficient prowess successfully.

The cascade model of training employed in our study is founded upon the principles of peer learning and is critical to the long-term goal of making breast cancer detection programmes in remote areas fully self-sustaining. The success of peer learning has been consistently demonstrated in various avenues of healthcare education, including clinical medicine and public health [20]. In our study, phase two was able to conclusively demonstrate the suitability and effectiveness of peer learning in educating and training HCWs. It was especially heartening to note that despite the learners in phase two (taught by MTs) having lower baseline knowledge and skills than the learners in phase one (taught by fellowship-trained breast surgeons), they were able to achieve comparable or even superior improvements across all domains compared to their phase one counterparts. Moreover, the success of phase two also demonstrated the replicability and scalability of the educational and training intervention, both of which are essential to expanding the training programme to involve more HCWs across GBC. Lastly, the continued proficiency in the HCWs’ CBE skills observed one year after the initial training confirmed the longevity of the training programme’s effectiveness. All the factors above strengthen the self-sustainability of the peer-learning-based cascade training model and can make early breast cancer detection available closer to home for rural and remote communities. This provides an opportunity to create breast care awareness, dispel myths and provide access to CBE at the community’s doorstep. This could serve as the first step in the detection of symptomatic breast lesions, enabling early referral, diagnosis, and treatment.

While our study lays the foundation for training and empowering a healthcare workforce for a sustainable community outreach programme, there remains the need for strengthening referral pathways for patients requiring further evaluation for breast abnormalities identified on CBE. Unfortunately, the development of such referral pathways is plagued by familiar problems, including a lack of transport infrastructure, specialised healthcare resources in the vicinity, inability to afford healthcare expenses, and cultural restrictions that limit women’s travel. Possible solutions may entail significant public-private partnerships and integrating an early breast cancer detection programme into existing governmental public health interventions such as the Lady Health Worker Program and Universal Healthcare Coverage [19–24] In addition, telemedicine initiatives may also be leveraged to negate geographical barriers to follow-up for clinical evaluations [25]. Moreover, based on our prior experiences with implementing large-scale early breast abnormality detection programmes in Sindh [13], it is critical to maintain strict records of patients’ compliance with referrals, further evaluation, and treatment if necessary.

Our study is not without limitations. First, although the HCWs demonstrated proficient CBE skills while undergoing DOPS, their performance may have been biased due to the Hawthorne effect. Thus, some level of quality assurance and monitoring must be introduced to ensure that HCWs perform CBEs with the same degree of accuracy and proficiency in the field. Second, it is important to understand the efficiency of HCWs in performing CBE within a community to better allocate time, resources, and personnel within the outreach programme. Third, as our study was not geared towards validating CBE in our setting, we cannot comment on the eventual rates of false negatives and false positives of using CBE as an early detection tool when performed at a mass level within the population. Additionally, including HCWs from all professional backgrounds, albeit more representative of a real-world setting, introduced a certain degree of heterogeneity in our results as different HCWs had varying prior knowledge regarding breast cancer and CBE. Moreover, the evaluation of the HCWs in the study was conducted by the same breast surgeons who delivered the education and training to the MTs, which may introduce a level of bias in our study. Also, while our study was adequately powered to detect a change in competency across the total group of HCWs, our sample size precluded us from performing extensive analyses. Lastly, we have yet to study the real-world, tangible clinical and oncologic outcomes of this outreach programme on the detection, evaluation, and treatment of breast cancer within the target population in Gilgit-Baltistan.

## CONCLUSIONS

In our study, we demonstrated that mass training of outreach HCWs in remote regions in performing CBE may be possible with a structured multiphase cascade-training model. Further exploring the scalability and replicability of such a programme is an important step in the early detection and downstaging of symptomatic breast cancer in geographically remote and low-resource settings. Future work must also seek to evaluate the impact of such a programme on the clinical and oncologic outcomes of breast cancer.

## Additional material


Online Supplementary Document
