# Supplementary material for: A multi-phase structured cascade model for mass training of community healthcare workers in performing clinical breast exams in remote regions
Source: J Glob Health. 2024 Dec 20;14:04255. doi: 10.7189/jogh.14.04255 (PMC11659790; doi:10.7189/jogh.14.04255)
Supplement: Online Supplementary Document [file jogh-14-04255-s001.pdf]

Online Supplementary Document

Appendix S1

Knowledge Assessment: (1) (2) نالج اسسمنٹ

Date: تاریخ ________________________________

Site: سائیٹ ________________________________

Name: نام. ________________________________

Test Type (circle on e) : ٹیسٹ کی قسم

Pre-test پری ٹیسٹ **Post-test** پوسٹ ٹیسٹ

**Mark Yes, No or Don't Know for the following:**<br>مندرجہ ذیل کے لیے ہاں، نہیں یا معلوم نہیں پر نشان لگائیں۔
1. Breast cancer is curable if detected early.<br>بریسٹ کینسر قابل علاج مرض ہے اگر جلدی پکڑا جائے۔<br>a. Yes — ہاں .a<br>b. No — نہیں .b<br>c. Don’t know — معلوم نہیں .c
2. Only individuals older than 50 years can get breast cancer.<br>بریسٹ کینسر صرف پچاس سال سے زیادہ عمر کے لوگوں کو ہوتا ہے۔<br>a. Yes — ہاں .a<br>b. No — نہیں .b<br>c. Don’t know — معلوم نہیں .c
3. Breast cancer only occurs in women.<br>بریسٹ کینسر صرف عورتوں میں ہونے والی ب<br>a. Yes — ہاں .a<br>b. No — نہیں .b<br>c. Don’t know — معلوم نہیں .c
4. A woman who has given birth to a child and has breast-fed cannot get breast cancer.<br>ایسی عورت جس نے بچہ پیدا کیا ہوا اور بچے کو دودھ پلایا ہوا اس کو بریسٹ کینسر نہیں ہو سکتا۔<br>a. Yes — ہاں .a<br>b. No — نہیں .b<br>c. Don’t know — معلوم نہیں .c
5 Needle biopsy of a cancerous breast lump can lead to spread of cancer.<br>چھاتی میں کینسر والے کٹھلی کی سوئی سے بائیوپسی کرنے سے پھیل سکتا ہے<br>a. Yes — ہاں .a<br>b. No — نہیں .b<br>c. Don’t know — معلوم نہیں .c

Choose the, best answer (circle only one)

6. How often should a woman have a clinical breast exam?

عورت کوکتنے وقفہ سے چھاتی کا معائنہ کروانہ چاہیے

a. Weekly
b. Monthly
c. Twice a year.
d. Yearly
e. Don't know

a. ہر ہفتہ
b. مہینےمیں ایک دفعہ
c. سال میں دومرتبہ
d. سالانہ
e. معلوم نہی

7. What is the most common cause of breast cancer in women?

عورتوں میں بریسٹ کینسریاچھاتی کےسرطان کی وجہ عام طورپرکیاہوتی ہے-

a. A family history/ genetic
b. Sporadic/ no clearly identifiable cause.

a. خاندان میں بیماری ہونا
b. وجہ نہ ہونا

8. Breast cancer is treated by

بریسٹ کینسر کا علاج اس ذریعہ سے کیا جاتا ہے

a. Surgery only
b. Chemotherapy alone
c. Endocrine therapy alone
d. Radiation alone
e. A combination of above

a. صرف سرجری
b. کیموتھراپی
c. اینڈو کرائین تھراپی
d. یڈیشن
e. اوپر کا مجموعہ

9. In a woman diagnosed with breast cancer, the surgical procedure includes:

عورت میں بریسٹ کینسر کی تشخیص کے بعد علاج میں اپریشن بھی شامل

a. Surgery on breast only.
b. Surgery on axillary lymph nodes.
c Surgery on both breast, and axillary lymph nodes

a. آپریشن صرف چھاتی پر
b. پریشن صرف بغل کے غدودا
c. پریشن چھاتی اور بغل کے غدودا

10. What is the next diagnostic step after a woman is confirmed to have a palpable mass in the breast?

ورت کی چھاتی میں گٹھلی کی تصدیق ہونے کے بعد تشخیص کیلئیے سب سے پہلے کونسا ٹیسٹ کروانا ہوگ

a. Breast imaging such as mammogram/ultrasound
b. Breast Surgery
c. Needle biopsy of breast

a. چھاتی کی امیجنگ جیسے میموگرام/الٹراساؤنڈ
b. چھاتی کی سرجری
c. چھاتی کی سوئی سے بائیوپسی

Select all that apply for the following (can choose multiple):

ین سب کو منتخب کریں جو درج ذیل کے لیے لاگو ہوتے ہیں (متعدد کا انتخاب کر سکتے ہیں)

11. Chance of getting breast cancer increases with:

کینسر ہونے کا امکان بڑھتا ہے جب

a. As a woman gets older
b. With family of history breast cancer
c. Not having given birth to a child
d. Obesity or increased weight
e. Using a brassier

a. عورت کی بڑھتی عمر
b. خاندان میں بریسٹ کینسر ہو
c. بچے کو پیدا نہ کرنا
d. موٹاپا یا بڑھتا ہوا وزن
e. یزئیرکا استعمال

12. Signs of breast cancer

بریسٹ کینسر کے علامات

a. Change in the position of the nipple — a. نپل کا رخ تبدیل ہونا
b. Pulling in the nipple — b. نبل کا اندر کھینچنا
c. Puckering or dimpling of breast skin — c. جلد میں گڑھے بڑھ جانا
d. A lump in the armpit — d. بغل میں گٹھلی
e. Redness of the breast — e. چھاتی کے جلد میں سرخی
f. A lump in the breast — f. چھاتی میں گٹھلی
g. Changes in the shape of the breast — g. چھاتی کے شکل میں تبدیلی

13. What does it mean to have a family history of breast cancer?

بریسٹ کینسر کی فیملی ہسٹری ہونے سے کیا مراد ہے

a. Having a mother who had breast cancer. — a. ماں میں بریسٹ کینسر
b. Having a sister who had breast cancer — b. بہن میں بریسٹ کینسر
c. Having a mother's or father's sister who had breast cancer — c. خالہ یا پھوپی میں بریسٹ کینسر
d. Having a cousin who had breast cancer — d. کزن جس کو بریسٹ ہو
e. Having a father who had breast cancer — e. باپ کو بریسٹ کینسر
f. Having a grandmother who had breast cancer — f. نانی یا دادی مین بریسٹ کینسر

Mark on a scale of 1 – 5
ایک سے پانچ تک کے پیمانے پر نشان لگائیں

14. How confident are you in your ability to detect a breast lump >2cm in size?

پ اپنی معائنہ کرنے کی صلاحیت کو جانتے ہوئے چھاتی میں ۲ سی ایم سے بڑی گٹھلی پکڑنے کے بارے میں کتنی پراعتماد ہیں ا

1	2	3	4	5
Not confident at all	Slightly confident	Somewhat confident	Fairly confident	Completely confident
بالکل نہیں	بہت تھوڑی	تھوڑی بہت	چھی خاصی	بہت زیادہ

References:
1. Keating NL, Kouri EM, Arreola Ornelas E, Magaña Valladares L, Marie Knaul F, on Mexicana para la Salud F, et al. Evaluation of Breast Cancer Knowledge Among Health Promoters in Mexico Before and After Focused Training. Global Health and Cancer The Oncologist [Internet]. 2014 [cited 2021 Dec 15];19:1091–9. Available from: http://dx.doi.org/10.1634/theoncologist.2014-0104
2. Dusengimana JMV, Keating NL, Hategekimana V, Rugema V, Bigirimana JB, Costas-Chavarri A, et al. Impact of Breast Cancer Early Detection Training on Rwandan Health Workers' Knowledge and Skills. Journal of Global Oncology [Internet]. 2018 Mar 1 [cited 2021 Dec 2];4(4). Available from: /pmc/articles/PMC6223427/

Appendix S2

Modified Direct Observation of Procedural Skill (DOPS) for Evaluation of Clinical Breast Examination Technique

Date: ____________________________________

Name: ____________________________________

Choose the appropriate answer for the following:
I. The person being evaluated is a:
a. CHW b. LHV c. LHW d. Nurse e. GP/gyn f. LMO
g. Other: ________________

II. Test Type:
Pre-test Post-test

III. Setting where evaluated:
Clinical Simulated Other: ________________

IV. The patient on whom the evaluation is conducted is:
a. N/A (simulator) b. Initial c. Follow-up

V. Patient's Diagnosis (if any established at the time of assessment):
a. None /normal b. Breast lump/other abnormality underwork-up
c. Breast cancer confirmed on biopsy awaiting surgery d. Breast cancer survivor (previously treated)
e. N/A

For each of the following, choose one (satisfactory/Unsatisfactory or Not Applicable):

Clinical Breast Examination Assessment				
		Satisfactory	Unsatisfactory	Not Applicable
1	Examined Sitting	1	0	
2	Examined Supine	1	0	
3	Examination of lymph nodes:			
	Cervical lymph nodes examined	1	0	
	Supraclavicular lymph nodes examined	1	0	
	Axillary lymph nodes examined bilaterally (2 points if both sides)	2	0	
	Was able to identify lymphadenopathy if present	1	0	N/A
Inspection				
4	Appropriately commented on breast symmetry	1	0	
5	Appropriately identified overlying skin changes if present (mark all that apply):			
	a. Cellulitis	1	0	N/A
	b. Edema	1	0	N/A
	c. Peau d'orange (orange peel texture of skin)	1	0	N/A
	d. Other visible skin changes, such as:<br>• Skin dimpling<br>• Deformity/ abnormal shape<br>• Visible nodules	1	0	N/A
6	Appropriately commented on nipple-areola complex (normal symmetry, retraction/ deviation, erosion)	1	0	
Palpation				

7	Appropriate technique used:<br><br>*Palpation done using the finger pads of the index/ middle/ fourth finger, in small concentric circles using either:*<br>o *Vertical stripe pattern*<br>o *Circular pattern*	1	0	
8	All areas of the breast were palpated?	1	0	
9	Able to identify palpable lump if present<br><br>*Difficulty of Case (to be decided by the assessor according to the level of examinee):*<br><br>• ☐ a. Must pick-up (lump>2cm, visible changes)<br><br>• ☐ b. Good to pick-up (small lump <2cm)	1	0	N/A
10	Inference			
	Overall assessment of the breast by the examinee :<br><br>• ☐ a. Normal (no visible changes or palpable abnormality)<br><br>• ☐ b. Abnormal (asymmetrical finding on either visual inspection or palpation warranting further work-up /referral)			
	Inference consistent with the assessor's	1	0	

Time taken to perform CBE: ____________

Comments (if any): ____

Assessor's Name and Designation:_____________

Assessor's signature: _______________

References:

Bates Guide To Physical Examination and History Taking 13th Edition - Dr Notes. (n.d.). Retrieved December 10, 2021, from https://dr-notes.com/bates-guide-to-physical-examination-and-history-taking-13th-edition-6er

Breast Exam | Stanford Medicine 25 | Stanford Medicine. (n.d.). Retrieved December 10, 2021, from https://stanfordmedicine25.stanford.edu/the25/BreastExam.html

Mcdonald, S., Saslow, D., & Alciati, M. H. (2004). Performance and Reporting of Clinical Breast Examination: A Review of the Literature. *J Clin*, *54*, 345–361. http://CAonline.AmCancerSoc.org

Bobo JK, Lee NC, Thames SF. Findings From 752 081 Clinical Breast Examinations Reported to a National Screening Program From 1995 Through 1998. JNCI: Journal of the National Cancer Institute [Internet]. 2000 Jun 21 [cited 2021 Dec 20];92(12):971–6. Available from: https://academic.oup.com/jnci/article/92/12/971/2905789
